# Justification of Galston’s liberal pluralism

**DOI:** 10.1186/s40064-016-2993-8

**Published:** 2016-08-08

**Authors:** Golam Azam

**Affiliations:** Department of Philosophy, Dhaka University, 2032, Arts Building, Dhaka, 1000 Bangladesh

**Keywords:** Multiculturalism, Liberal pluralism, Mutualism, Toleration, Galston, Historical injustice

## Abstract

Liberal multicultural theories developed in late twenty-first century aims to ensure the rights of the minorities, social justice and harmony in liberal societies. Will Kymlicka is the leading philosopher in this field. He advocates minority rights, their autonomy and the way minority groups can be accommodated in a liberal society with their distinct cultural identity. Besides him, there are other political theorists on the track and Galston is one of them. He disagrees with Kymlicka on some crucial points, particularly regarding the scope of civil rights of the minority groups and the responsibilities of both majority and minority groups for the sake of social harmony and justice. He tries to develop a moral theory of mutualism based on inter-community toleration and constitutionalism. Considering toleration as one of the fundamental liberal virtues he contends that the majority government has the responsibility to make arrangement both for the members of majority and minority groups so that they can build inter personal relation and learn toleration. The paper critically justifies the feasibility of his theory in a liberal society and claims that although Galston’s theory has a higher possibility to be accommodated in liberal societies, it eventually fails.

## Background

In discussing how liberal-democratic States ought to relate to non-liberal cultural minorities within their jurisdiction, liberals are divided into two camps. Some liberals argue that the liberal State should be maximally tolerant of different ways of life even of those that are deeply illiberal. Other liberals think that liberal democracy is itself the political expression of a valuable way of life with a distinctive set of values and virtues and that the liberal state is entitled, even obliged, to encourage its non-liberal minorities to accept liberal values in order to protect the cultural underpinnings that are essential to the flourishing of liberal political institutions.

Galston argues for the priority of toleration over individual autonomy in the liberal schedule of values (Crowder 2004). According to him, the problem with autonomy-centric liberalism is that it stands for one particular understanding of the human good, and so is too narrow for the legitimate diversity of lives to be found in a modern society. That diversity includes outlooks that ‘cannot conscientiously embrace the Enlightenment impulse’ (2002, pp. 25–26). An attempt to use State power to promote individual autonomy can weaken or undermine individuals and groups that do not and cannot organize their affairs in accordance with that value without undermining the deepest sources of their identity (Galston 1995, p. 525). Autonomy-centric liberalism not only fails to properly respect individuals with non-liberal ideals, Galston argues, if liberals insist on promoting the ideal of autonomy in all spheres of society, they risk alienating ‘many citizens of goodwill’ and creating opponents in place of allies (2002, p. 26). In this paper I argue in favor of my claim that since Galston’s theory provides a promising approach to social and political justice, peace and human rights in a plural society, his liberal pluralism has a higher potentiality to be a guiding political theory in liberal society.^1^ I also defend my other claim that the theory eventually fails.

## Galston’s theory of liberal pluralism

Galston’s theory of multiculturalism encompasses his conception regarding diversity, expressive liberty, toleration and mutualism within a liberal framework. Galston says, “by ‘diversity’ I mean, straightforwardly, differences among individuals and groups over such matters as the nature of the good life, sources of moral authority, reason versus faith, and the like” (2002, p. 21). He characterizes diversity in a liberal society as a fact, as an instrumental value and as an intrinsic value.

Galston says that we must acknowledge wide diversity as a fact that can be significantly altered only through the employment of unacceptable degrees of State coercion and with unacceptable levels of civil strife. Using Mill’s (see Mill 1977) celebration of individuality, diversity can be defended as having instrumental value by providing individuals with many different examples of how to achieve a satisfying life. But diversity can also be regarded as an intrinsic value. For Galston, our moral universe is characterized by plural and conflicting values that cannot be harmonized in a single comprehensive way of life and a wide range of such goals and conceptions could serve as bases of worthwhile life (2002, p. 27).

Galston’s pluralism derives from his acceptance of Berlin’s notion of value incommensurability: human goals are many, not all of them are commensurable (Berlin 1969, p. 171). According to Berlin, “The world that we encounter in ordinary experience is one in which we are faced with choices between ends equally ultimate and claims equally absolute, the realization of some of which must inevitably involve the sacrifice of others”(1969, p. 168). Hence there is no single ultimate right answer to questions of value; rather a plurality of right answers derivable from a plurality of values that are equally ‘ultimate’ (Crowder 1994, p. 294). Galston’s multiculturalism is based on the belief that our moral universe is characterized by plural and conflicting values that cannot be harmonized in a single comprehensive way of life. Nor can they be reduced to a common measure or ranked in a clear order of priority.

If the moral philosophy of pluralism is roughly correct, there is a range of indeterminacy within which various choices are rationally defensible though not rationally required. Given that no one uniquely rational ordering or combination of incommensurable values exist, no one could ever provide a general valid reason, binding on all individuals, for a particular ranking or combination. Reflective policies, whose justifications includes the assertion that there is a unique rational ordering of value, therefore have no basis (Galston 1998a, p. 886).

Nevertheless, moral pluralism is not a form of relativism. Galston insists that there are things that are objectively bad, such as the great evils of human existence, and they should be rejected both in individual and collective life. Contrarily, there are things, which are objectively good, such as respecting others.

Galston maintains that “properly understood liberalism is about the protection of diversity, not the valorization of choice” (1991, p. 329). In a liberal multicultural society, according to his view, intrinsic goods are not all political goods. There are heterogeneous goods: private, social, familial and even religious. Although these goods can affect the political order, they do not exist for the sake of politics (2002, p. 38). Moreover it is not reasonable to suppose that political goods always enjoy a comprehensive priority over others in every context. “Moral pluralism lends support to the proposition that the state should not be regarded as all-powerful, while political pluralism helps define and defend the social space within which the heterogeneity of value can be translated into a rich variety of worthy human lives” (2002, p. 38).

He allows that the capacity of a liberal political order to accommodate multiple conception of the good life is limited. Liberalism can legitimately reject some conceptions of good. Following Berlin’s notion of the ‘common moral horizon’, he thinks that certain very basic values must always be respected if a given society is to count as minimally decent (Galston 1999, 2002, p. 50). This account of what must be accepted in a liberal society has implications for the relation between religion and politics. Some faiths do endorse a clear hierarchy of binding values; some are against free expression. On the other hand some faiths are characterized by internal value pluralism and are predisposed to accept a pluralist society. Galston also thinks that there are zones where value pluralism and religious beliefs overlap. In such situation he contends, “once the multiplicity of faiths is an irreversible fact, other considerations –many themselves faith-based–come into play to restrict state coercion on behalf of any single faith. This is a kind of restraint on certain religious practices, and it may well stack the deck in favour of faiths that emphasize inward conscience rather than external observance” (1998b, p. 243). Galston thus contends that while autonomy poses clear challenges to faith, the moral philosophy of value pluralism straightforwardly hospitable to faith either. For example, restricting polygamy for the Muslim men in a liberal country. However, Galston holds that value pluralism establishes a meaningful social space for religious belief and practices i.e. at least those who are willing to practice religious beliefs, have the space to do so.

A pluralist society not only permits and encourages the existence of plural goods. It also has multiple sources of authority—individuals, parents, civil associations, faith-based institutions, and the state, among others—no one of which is dominant in all spheres, for all purposes, on all occasions (Galston 2004, p. 36). Galston contends that

Pluralist politics is a politics of recognition rather than of construction. It respects the diverse spheres of human activity; it does not understand itself as creating or constituting those activities ex nihilo. Families are shaped by public law, but that does not mean that they are wholly socially constructed. There are complex relations of mutual impact between public law and faith communities, but it is preposterous to claim that the public sphere creates these communities (2006, p. 827).

He also considers that “as so many types of human association possess an identity not derived from the state, pluralist politics does not presume that the inner structure and principles of every sphere must mirror those of basic political institutions” (2004, p. 38). For example, in order to fill positions of religious authority, religious communities may use, without state interference, gender-based norms that would be forbidden in businesses and public accommodations.

### Expressive liberty

Because liberal societies allow maximum scope for diversity, they are most conducive to the development of individuality. And because liberal societies rest on individual freedom, they tend to foster the self determination that is at the heart of true individuality (Galston 1988). In a liberal society it is often supposed that autonomy and diversity complement each other but, “… these principles do not always, or usually, cohere” (2002, p. 21). Rather sometime they create conflict in different area of practical life such as education or free exercise of religion. For example, in a liberal country an illiberal group may not allow their girls to go to school after a certain age whatever is the desire of the girls. Therefore, conflict arises between individual autonomy and a form of diversity that allows restriction.

Galston’s idea of liberty i.e. ‘expressive liberty’ is thus not the same as autonomy. By expressive liberty he means, “… the absence of constraints, imposed by some individuals on others, that make it impossible for the affected individuals to live their lives in ways that express their deepest beliefs about what gives meaning or value to life” (2002, p. 28). Those who exercise expressive liberty may not value autonomy. “It [expressive liberty] protects the ability of individuals and groups to live in a ways that others would regard as unfree” (2002, p. 29). Galston thinks that expressive liberty is a human good that all societies ought to protect because its absence is an occasion for misfortunes that few would willingly endure (2002, p. 29).

In contrast to the concept of autonomy, expressive liberty demands that individuals be free to identify with their aims and projects, not that they should think of them as dictated by reason or as being the best choice among alternatives. Expressive freedom can be possessed by an individual who lives in conformity with religious tradition and obeys what she regards as divine commands, given that it is her beliefs about the good that direct her life and not coercion by her parents or her church. Expressive freedom does not have to be realized through free choice. For Galston,

Not all sets of practices will themselves rest on, or reflect a preference for, liberty as ordinarily understood. For example, being Jewish is not always (indeed is not usually) a matter of choice. But once that fact is established through birth and circumstance, it becomes a matter of great importance for Jews to live in a society that permits them to live in accordance with their understanding of an identity that is given rather than chosen, and that typically is structured by commandments whose binding power does not depend on individual acceptance (1998a, p. 877)

Since it is expressive liberty rather than autonomy that is central to a liberal pluralist State, this must be reflected in its institutions and the education it gives to it’s citizens. A liberal pluralist government may legitimately engage in civic education but this must be carefully restricted to the virtues and competences that citizens need to fulfill diverse roles in a liberal pluralist economy, society, and polity. A liberal pluralist State has a legitimate and compelling interest in ensuring that the convictions, competences, and virtues required for liberal citizenship are widely shared. Expressive liberty is possible only within societies whose members do not impede one another’s opportunity to live their lives as they see fit (Galston 2002, p. 28). Galston contends, “Expressive liberty has civic preconditions—in particular, internalized norms of self-restraint when faced with practices that reflect understandings of the good life one does not share. Fostering this self-restraint is (within limits) a legitimate object of State action” (2002, p. 29).

### Toleration

Galston thinks that a central task of the liberal State is ‘accepting and managing diversity through mutual toleration’. The maintenance of a society that accepts diversity and expressive freedom requires the virtue of toleration. This virtue does not require the belief that every personal choice, every life-plan, is equally good, and hence beyond rational scrutiny and criticism. Toleration is fully compatible with the conviction that some ways of life are superior to others. It does not require an easy relativism about the good. It is compatible with engaged moral criticism of those with whom one differs. Toleration means, rather, a principled refusal to use coercive power to impose one’s own views on others, and therefore a commitment to moral competition through recruitment and persuasion alone (Galston 2002, p. 126). Indeed, toleration may be defined as the ability to make this conviction effective as a maxim of personal conduct (1988, p. 1282).

Toleration is conscientious reluctance to act in ways that impede others from living in accordance with their conception of what gives life meaning is necessary for expressive liberty. It is a precondition of a society which is able to accommodate diversity limited only by the minimum requirements of civic unity. But citizens cannot be expected to be naturally tolerant. It is thus important that the government of a liberal society take the initiative to develop it through education and in civic life. Since it is hard to believe that toleration, so understood, can be cultivated without at least minimal awareness of the existence and nature of ways of life other than those of one’s family and community, the State may act to ensure that people acquire this knowledge. What it may not do is prescribe curricula or pedagogic practices that aim to make students skeptical or critical of their own way of life (Galston 1995, p. 127). He thinks that civic education conducted in a multiculturalism spirit will be robust but carefully restricted to essentials (2002, p. 126).

### Mutualism

One of the fundamental challenges in a multicultural liberal democracy is discovering a way to tie people of different faiths and ethnicity together as members of a common society.^2^ In Galston’s theory this is accomplished by what he calls mutualism. By ‘mutualism’ he means a moral outlook that underpins a society as an association of individuals for mutual advantage, along with an understanding of mutual responsibility—individual and social—that flows from this conception (2003, p. 212). Mutualism supports political and economic institutions to which citizens contribute if they can and social provision for those who cannot. It endorses equality of opportunity that is more than formal but does not require equal outcomes. It promotes a limited but robust sense of shared fate. According to Galston, the idea of mutualism also insists that individuals are linked through a dense network of natural duties and that societies exist in part to give force to these duties. Human beings are both separated and connected, and any viable conception of human society must give due expression to both these aspects of our existence (2005, p. 167). According to him if both individual and collective responsibility are understood broadly, we get a State whose guiding principle is reciprocity. This principle is the heart of the mutualist alternative.

Galston thinks that within a pluralist framework securing a viable political community must satisfy preconditions: there must be public order of a minimum kind, the rule of law and common membership in a political community. For Galston, public order of a minimum kind must be maintained. Pluralists recognize that anarchy is the enemy of pluralism. To have a stable society is it necessary to have and enforce stable property relations. The rule of law must prevail and the economy must not divide the population permanently between a thin stratum of the rich and the numerous poor. Citizens must have a sense of membership in their political community strong enough to override ethical and religious differences (Galston 2002, pp. 65–66). Galston holds that public institutions are necessary to create a secure space within which individuals and groups may lead their lives in accordance with their diverse understanding of what gives life meaning and value and that it is justified for the government and citizens to ensure that this space exists.

Like Rawls (1993, 1999) Galston believes that a viable political society requires a shared conception of justice. But this shared conception must be as accommodating as possible. The liberal pluralist notion of justice has four basic elements: (a) the rule of the law, (b) equal citizenship (c) public dialogue for determining the issues of equality, need, choice etc., and (d) enjoyment of social minimum as far as resources are concerned.^3^ How these elements should be spelled out depends on a particular polity and its history. For example, a particular political society may decide to guarantee to individuals more than the social minimum. But “there is no general theory that obliges particular communities to resolve such matters in a uniform fashion; there is wide scope for legitimate variation, guided by public preferences articulated in public choices. Liberal pluralist justice shapes politics not replaces it” (Galston 2002, p. 128).

In a mutualist multicultural society citizens have equal rights and responsibilities. They not only have a duty to uphold the law. They must also contribute to fulfilling the purposes for which the society was formed. But Galston wants to make room for groups that do not share this common purpose—so long as they are willing to obey the law. He says that

…the stress on shared citizenship as the basis of public norm enforceable against groups raises the possibility of some intermediate status … for groups that are willing to abide by the basic laws of the community without making full claims upon it, in return for which they might be exempted from some requirements of full citizenship (2002, p. 127).

It is permitted for members of groups to withdraw substantially from the civic community in order to live out a distinctive vision of the good life shared by few others, so long as this vision does not involve gross violations of human rights like slavery or human sacrifice (Spinner-Halev 1999, pp. 65–86). Galston thus allows for groups who reject liberalism altogether—though they too are required to have a minimum civic education that encourages toleration and mutual respect.

## Justification of Galston’s liberal pluralism

There are two untwined aspects of Galston’s pluralism. On the one hand as a positive aspect it seems to have a higher capability to be a potentially viable theory in liberal countries to lessen injustice and uphold the rights and status of the minority groups through developing inter-personal relation and mutualism based on toleration and respect. In this perspective Galston’s theory is in a better position than that of Kymlicka. However, there are at least two crucial limitations of the theory, which make it vulnerable in getting acknowledgment as a potentially most promising theory of pluralism.

### Analysis of the first claim

First claim that is to be sought to analyze here is how Galston’s pluralism is more accommodative and have higher potentiality to ensure stability in a liberal society than Kymlicka. Kymlicka (1995) in his liberal multiculturalism prescribes various kinds of rights for minority groups without abandoning liberal values of liberty, equality, toleration and government neutrality. He thinks that a liberal society should promote autonomy and that non-liberal cultures should be encouraged to value individual choice and rational critique. He also argues for the differentiated rights of the minorities of different types; natural minorities, ethnic minorities and immigrant minorities. He contends that justice can be prevailed if the autonomy of individuals can be respected as well as everyone’s culture-bound share can be distributed fairly. However, Kymlicka’s theory has its weaknesses. For one thing, his emphasis on the value of individual autonomy makes it difficult for his theory to adequately deal with groups that do not value individual autonomy.

Galston contends that a liberal State that valorizes autonomy cannot properly accommodate plurality. Accommodating pluralism requires accommodating those who do not value autonomy. As in the case of all advocates of liberalism, there are limits to what he thinks a liberal State can tolerate. But for him the purposes of liberal State do not include the promotion of autonomy or of more egalitarian forms of equality. The purpose of protecting human life and the normal development of basic physical and psychological capacities give it an entitlement to intervene in practices that threaten individuals with serious harm. The task of ensuring that citizens are able to participate in the society, politics and economy give it the justification for promoting forms of education that enable ‘social rationality’ and prohibiting those that undermine it. But fulfilling these purposes would not justify a liberal State in interfering with a group simply because it does not value individual autonomy or subscribe to egalitarian ideas of justice. Galston thinks liberalization is likely to turn out to be the cultural equivalent of the Vietnam-era principle of destroying the village in order to save it (2002).

Galston also rejects another controversial aspect of Kymlicka’s theory regarding differential rights for communities of different kinds. He is not interested in the reasons why groups want to adhere to their values or how groups came to be in the country in the first place. What is important to him is simply that people do have diverse values that are centrally important to their lives. On the other hand he is centrally concerned with how people with these diverse values can live together in a political society. His emphasis on civic education and toleration are important aspects of his multiculturalism.

The beauty and distinctness of Galston’s pluralism is that it speaks about expressive liberty rather than mere autonomy, it speaks about toleration as founding principle for social and political stability in a plural society, it urges for the promotion of liberal virtue and unknown virtues lying in non-liberal community through education and associations both institutional and non-institutional ways, and above all it tries to reconcile between duties and responsibilities of every citizen irrespective of her cultural identity. Galston also thinks that there could be something alternative to democracy. Such an idea is novel and an addition to any other theory of multiculturalism. According to Galston, mutualism could exist in a non-democratic country providing that toleration and the rule of law prevails. Galston, centrally concerned with citizenship in a diverse society and how toleration can be promoted, advocates a liberal society that is capable of encompassing diversity—including people and groups who do not value autonomy.

### Analysis of the second claim

Although Galston’s pluralism has a higher potentiality to be accommodated in a liberal society, it has two major limitations among others because of which it becomes vulnerable to be the most promising theory of multiculturalism. Two such problems are its failure to address historical injustices and to adequately secure political participation of minority groups.

The issue of historical injustice is one of the major factors of social anarchy. ‘… redressing past wrongs is essential to establishing conditions of justice in a society scarred by the enduring and pervasive effects of those wrongs’ (De Greiff 2003; Torpey 2003). The past injustices done to the minority individuals and groups are mainly political violence, an institutional way of undermining the equal worth of persons because of their ethnicity, religion, culture or political belief and action. As a person or victim she can be targeted more than one of these characteristics at once (see Jones 2004, pp. 2–10). Such wrongdoing as political violence disrupts the fundamental moral premises of a liberal democracy because liberal democratic institutions have to ensure that citizens are treated as persons with equal moral worth. According to Rawls, a liberal democracy should be understood as a system of social cooperation between free and equal persons (Rawls 1971). Thus respect for people’s moral equality is important as an intrinsic feature of a system of cooperation for it to count as liberal and democratic. It is to be mentioned that being a victim of political violence has severe effects on people’s capacity to achieve a healthy psychological adjustment as well as to develop a life plan successfully. It might have similar severe affect on victimized groups. Such psychological state is termed as “political trauma”. This psychological trauma sometimes ended as non-cooperation, distrust, unwillingness to be accommodated, and isolation from the mainstream public life. Such past wrongdoing also demoralize the self-respect of persons which eventually impact on respect for others and mutual respect.

Study reveals that testimony is one of the ways in helping the act of psychological recovery from this political trauma. Hamber (2009), working on the post-apartheid cases in South Africa contends that programs, objects and actions of reparation in general have impact on citizens’ capacity for achieving a healthy psychological adjustment in the aftermath of political violence. These reparations seem to go beyond testimony:

The integral importance of reparations, remorse, restitutions, truth and acknowledgment to victims […] I have found that reparations by victims and survivors in process aimed at achieving such elusive goals as truth and justice is an important component of healing—many survivors want to feel they are taking some actions, even if they know it will not deliver complete justice or absolute truth. This gives survivors some control over their environment, something which political trauma normally overrides (Hamber 2009, p. 194).

By considering the importance of the rectification of historical injustice in such empirical way, there might have sufficient reasons to think such past injustice as one of the bases of individuals’ deficit in self-respect. Rawls refers to the social bases of self-respect as ‘perhaps the most important primary good’ (Rawls 1971, p. 386). This self-respect have four elements which make the person as a moral worth; a positive evaluation of herself, valuing her equal moral status with regard to the rest, the person must try to realize the system of ends that she adopts, and non-devaluation of her equal moral status and her own life plan by others (Vaca Paniagua 2012, p. 30). In case if one of these elements is agitated, her self-respect falls in deficit.

It is considered that a ‘past’ of a State shaped by political violence endures an extremely high importance to citizens and liberal societies particularly in relation to citizens’ psychology and self-respect, their mutual respect, and society’s liberal integrity as well as the achievement of social stability. Historical injustice not only affects victims’ psychology and self-respect but also the mutual respect that citizens owe to each other in social interactions. In this sense, a historical wrongdoing follows the description given by Thomas Scanlon regarding two different ‘evils’ that an unjust inequality might create. He says,

It is an evil to be treated as inferior” […] The experiential evil involved here can be characterized in several different ways […] Let me distinguish two broad categories. The first, more “individualistic”, characterization emphasizes what might be called damage to individuals’ sense of self-worth: such things as feelings of inferiority and even shame resulting from the belief that one’s life, abilities and accomplishments lack worth or are greatly inferior to those of others. The second category emphasizes damage to the bonds between people: what might be called the loss of fraternity resulting from great differences in material circumstances, accomplishments and the social importance according to them.(Scanlon 2003, p. 212)

Scanlon’s description of evils produced through unjust inequality is quite significant. While the first evil relates to the damage perpetrated on the self-respect of the person affected by the inequality, the second does the same to the relation between that person and the rest. According to the dependency thesis of Bird it can be assumed that deficit in mutual respect is conditioned with self-respect i.e. if there is deficit in self respect there will be deficit in mutual respect (Bird 2010, p. 17). The deficit in mutual respect eventually affects social integrity and social cohesion in a liberal country. Therefore historical injustice has to be rectified in an acceptable manner e.g. recognition, retribution, compensation and verbal apology etc.

Galston has little to say about the resentments that sometimes affect group relationships. Diversity in a plural society is caused not merely by differences of value. It can also be caused by historical factors—particularly by events that are perceived by some groups as an injustice committed against them (Thompson 2002; Spinner Halev 2007, 2001). We have seen that Galston’s theory tries to promote toleration, expressive liberty and mutual interrelation. However, among these elements of his pluralism, mutualism is the most promising. The fundamental basis of his mutualism is mutual respect, mutual interest and interest for the whole community. Non-rectification of historical injustice not only creates divisions but makes mutualism difficult to achieve also. People, suffered from oppression are not likely to think that they share common purposes with their oppressors. Moreover, such mutualism is in question when the issue of practical implication is taken into consideration. In societies, liberal or not, where there are ample of examples of historical injustices, rectification of such injustices might be considered as a precondition for the establishment of social solidarity and justice. In a pluralist society, any theory of plurality or theory of minority rights need to address properly the issue of historical injustices done to the minority groups in question, however, in some cases immigrant minorities might not face such trauma. According to Streich, an apology is one way to create a polity in which black Americans are equal moral, civic, and political members from the start and to recognize that this equality was denied as the founding of the US and delayed from reconstruction to the present”(Streich 2002, p. 541). Civic education, as Galston describes it, does not seem sufficient to heal wounds that have been caused by history.

Another problem of Galston’s pluralism ponders in giving minority groups’ access to decision-making body i.e. in politics. Though Galston advocates a plural society in which groups can live according to their cultural and religious values, his conception of politics is not much different from that of liberals who advocate common citizenship. Full citizens are individuals with a common political standing. They have an equal responsibility for fulfilling liberal purposes and have equal rights as citizens. Galston allows for groups that don’t want to participate in this way. But his mutualism assumes that these groups are very much in a minority. Like classical liberals he seems to assume that group allegiances, including religious belief, belong to private, or non-political, life and that in their political existence citizens participate as individuals. A mutualist State is a secular State and religion does not have a place in politics. This goes against the stance of those who think that religion should play a direct role in politics. But it also leads to a tension in Galston’s theory between giving groups such a large role in social life and no official role in politics. Minority groups in a liberal plural society, being deprived of a number of civil and political rights, often demand that the group itself should be represented directly in politics. The demand is, however, justified in some respects. For example, it’s problematic for the minority groups that it’s the majority people who decide about the life style of the minority group-members. For example, Jews, a minority group in USA, wants to close their shop on Saturday. Now if the majority who is not by religion Jews adopts a public policy that Saturday is not a weekly holiday, then its difficult for the minority Jews community to perform their religious and cultural commitment as a member of that particular group. Likewise, Muslims in USA requires holidays on their religious festive that they are accustomed in their Muslim majority homeland but it’s the majority who can only decide whether holiday can be announced on those days. As a result, it’s difficult for them to observe the festival on that particular day because as there is no representative from the community in decision-making body, the significance of the festive and its impact on the group-members remain unfelt by the majority groups. In the same line in a plural society, if it’s the majority who always take decisions about the minority ethnic or religious groups then it might fail to do justice to the minority groups. Moreover, like any other theory of liberal pluralism or multiculturalism, Galston’s theory is also not different form accepting majoritarian democracy. In a majoritarian democracy the minority groups have only option to be marginalized (Andeweg 2000; Arend 1986; Lijphart 1969). Galston speaks about many issues related to the interest of the minority groups such as expressive liberty, associationalism, mutualism, majority toleration and so. However, the question arises whether the majority group tolerates the participation of a minority representation in decision-making body with the power to veto to any policy subservient to their interest. Although this tension is not a serious matter in a society where secularism is accepted and where individualism is dominant. But it is bound to pose a problem in societies where group membership is crucial in political as well as social life and secularism is not widely accepted.

The third problem of Galston’s pluralism concerns its success in accommodating non-liberal groups in a liberal setting. The problem is based on the objection that there are ‘lacks of clarity about what a liberal State can tolerate and what it cannot’ in Galston’s theory. Galston makes the point that his theory of liberal pluralism cannot tolerate a gross violation of human rights even when group members regard such violations as part of their cultural life. For example, human sacrifice is not allowed even if those who are sacrificed accept this as a cultural norm (Galston 2002). The Aztec religion is out of bounds. Following Berlin, Galston argues that some highly generic norms are universal in the sense that they are mandatory for any form of life to count as ‘minimally human, decent, and morally acceptable’. But Galston offers no systematic account of the contents of the common moral horizon. Human sacrifice is an extreme example and we are left to wonder about other practices that are not so extreme but are nevertheless widely condemned. Would genital mutilation of women be allowed in a liberal society? Would it be allowable for a cultural group to severely punish homosexuals? Moreover, Galston himself points out that religious group that allows for differences of opinion and accepts diversity will have an easier time accepting the expressive liberty of others. But people who believe that their religion is the true religion and have a strong commitment to their beliefs will have difficulty tolerating practices that go against their beliefs. The Hindus consider “Cow” as a goddess and they respect Cow in as such. Therefore it is very difficult for them to tolerate a situation in which someone from a different faith slaughters a Cow. Accepting others’ right to have different beliefs is easier than tolerating practices that hurt people’s sacred and holy feelings. Such problems are more serious than some issues of moral debate e.g. the opposition between pro-life people who think that abortion is murder and pro-choice people who think that it is not, because in such moral debates the choice is personal but in cases mentioned above the opposition is connected to a group tradition and way of life. Galston’s mutualism depends heavily on toleration and toleration requires self-restraint. Indeed toleration that underwrites a society in which people are prepared to work together to maintain basic structures, defines a common concept of justice and to fulfill liberal purposes is likely to require more than a willingness to live and let live. Mutual respect seems to be required. Some critics therefore wonder whether non-liberal groups are likely to accept the kind of toleration that mutualism requires (Crowder 2004).

## Conclusion

The liberal multiculturalism of Galston fails to do justice to minorities at least in two respects. One of them is their failure to deal with the problem of historical injustice and the other is the problem of politics. Liberal multiculturalism provides an account of justice for minority groups. But it pays little attention to practical politics: how minority groups can ensure that a government will treat them justly. Practically speaking, having political power over crucial decisions is the only guarantee (at least in the opinion of people in many minority groups). Galston thinks that groups should be able to make their own decision about some matters, but they have typically liberal ideas about citizenship. Citizens are individuals. Participation in minority group decision-making is something apart from participation in decision making of governments. Groups themselves do not participate in political decision making of the State. This means that minority groups can still be at a disadvantage if political decisions go against them—if the State is not prepared to satisfy liberal multicultural requirements of justice. For example, Aborigines are a small minority in Australian population and if the government decides on a policy that disadvantages them, there is not much they can do about it. Having a few seats in a parliament, without veto power, dedicated to minority groups is not sufficient, because the majority can outvote these few representatives. Civic education might help—but probably not enough in a society where there is a lot of distrust and entrenched prejudices.

## Footnotes

1 Galston himself did not use this term for his theory. However, I think his theory counts as a theory of liberal multiculturalism. It is liberal because, as we will see, it subscribes to liberal values. It is multicultural because it aims to show how diverse cultures and communities can exist within a liberal State.

2 By “liberal democracy” Galston means a form of political organization in which individual and associational rights limit the scope of legitimate governmental authority and the powers of democratic majorities.

3 Liberal pluralists are committed to a conception of need that allows individuals to ward off the great evils of the human condition; what counts is relative to specific public purposes to which individual political communities give priority at particular moments in their history; every community builds an elements of choice or exchange into its understanding of distributive justice, and every community embeds choice within publicly defined limits; it is communities which must decide how to define the claims of equality with broader limits, however, from a liberal pluralists point of viewmembers of community must enjoy an equal social minimum that allows them to meet basic needs and participate in the activities of citizenship (Galston 2002, pp. 129–131).
